# Health-related quality of life in rare bleeding disorders: results from the Rare Bleeding Disorders in the Netherlands study

**DOI:** 10.1016/j.rpth.2025.102961

**Published:** 2025-06-27

**Authors:** Sterre P.E. Willems, Marjon H. Cnossen, Nick van Es, Paul L. den Exter, Ilmar C. Kruis, Karina Meijer, Laurens Nieuwenhuizen, Joline L. Saes, Nicole M.A. Blijlevens, Waander L. van Heerde, Saskia E.M. Schols

**Affiliations:** 1 Department of Hematology, Radboud university medical center, Nijmegen, the Netherlands; 2 Hemophilia Treatment Center, Nijmegen–Eindhoven–Maastricht, the Netherlands; 3 Department of Pediatric Hematology and Oncology, Erasmus MC Sophia Children’s Hospital, University Medical Center Rotterdam, Rotterdam, the Netherlands; 4 Department of Vascular Medicine, Amsterdam UMC location University of Amsterdam, Amsterdam, the Netherlands; 5 Amsterdam Cardiovascular Sciences, Pulmonary Hypertension and Thrombosis, Amsterdam, the Netherlands; 6 Department of Medicine-Thrombosis and Hemostasis, Leiden University Medical Center, Leiden, the Netherlands; 7 Dutch Association of Hemophilia Patients, Nijkerk, the Netherlands; 8 Department of Hematology, University Medical Center Groningen, Groningen, the Netherlands; 9 Department of Hematology, Máxima Medical Center Eindhoven, Eindhoven, the Netherlands; 10 Center for Benign Haematology, Thrombosis and Haemostasis, van Creveldkliniek, University Medical Center Utrecht and University Utrecht, Utrecht, the Netherlands; 11 Enzyre BV, Novio Tech Campus, Nijmegen, the Netherlands

**Keywords:** blood coagulation disorders, hemorrhagic disorders, menorrhagia, patient reported outcome measures, phenotype, quality of life

## Abstract

**Background:**

Clinical bleeding phenotype varies substantially among patients with rare bleeding disorders (RBDs). Patient-reported outcomes may provide valuable insights into health-related quality of life (HRQoL) and disease burden.

**Objectives:**

To evaluate HRQoL in patients with rare coagulation factor deficiencies and fibrinolytic disorders included in the nationwide, cross-sectional Rare Bleeding Disorders in the Netherlands (RBiN) study.

**Methods:**

Bleeding scores (ie, the International Society on Thrombosis and Haemostasis Bleeding Assessment Tool [ISTH-BAT]) were assessed during a single study visit, and electronic questionnaires captured demographic and HRQoL data (36-item Short Form survey [SF-36], Patient-Reported Outcomes Measurement Information System, Profile 29 [PROMIS-29]). Only differences exceeding the minimally important difference were considered clinically relevant and reported.

**Results:**

HRQoL data from 167 adults and 34 children were available. HRQoL of patients with RBDs measured by SF-36 was not significantly different compared to the Dutch reference population. PROMIS-29 scores indicated significantly better sleep, social participation, and pain-related outcomes in patients with RBDs than the reference populations. Subgroup analyses within the RBiN population showed worse physical health in patients with a severe bleeding phenotype than in those with a mild-to-moderate phenotype. Women with a history of heavy menstrual bleeding reported worse physical health and pain-related outcomes than those without. Patients reporting severe disease had worse pain interference and mental health scores (PROMIS-29) than those reporting nonsevere disease. ISTH-BAT scores were negatively associated with physical functioning.

**Conclusions:**

Overall HRQoL in patients with RBDs was comparable to the Dutch reference population. Within the RBiN population, a history of heavy menstrual bleeding, clinical bleeding phenotype, patient-reported disease severity, and ISTH-BAT scores were associated with impaired HRQoL, reflecting disease burden in patients living with RBDs.

## Essentials

• Living with a rare bleeding disorder (RBD) may impact health-related quality of life (HRQoL).
• HRQoL was assessed by SF-36 and PROMIS-29 questionnaires and compared to reference populations.
• HRQoL in patients with RBDs is at least comparable to Dutch reference populations.
• Heavy menstrual bleeding, bleeding severity, and patient-reported severity are associated with disease burden.

## Graphical abstract

**Figure:**
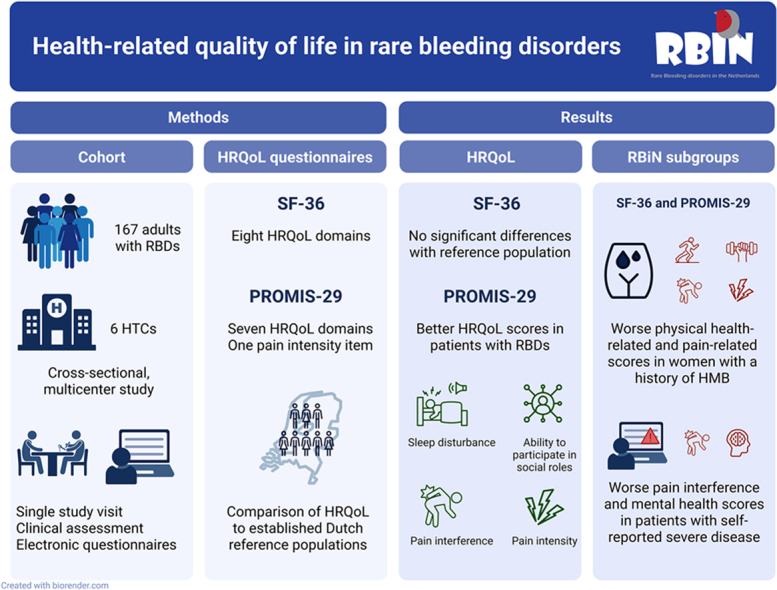

## 1 Introduction

Patients with rare bleeding disorders (RBDs) display a variable bleeding phenotype. Traditionally, RBDs encompass all coagulation factor deficiencies except hemophilia A and B. More recently, other RBDs such as disorders of fibrinolysis have been added to this definition [1]. Due to their rarity, RBDs are often studied as a group; however, they each represent distinct disease entities with varying clinical presentations [[1], [2], [3], [4], [5], [6]]. Bleeding severity ranges from asymptomatic to life-threatening, and frequently includes mucocutaneous, provoked (trauma/surgery), and sex-specific bleeding [[7], [8], [9], [10]].

Although RBDs are generally considered to be autosomal recessive, a heterozygous state does not necessarily imply a disease-free state [11]. Common classifications of disease severity—based on coagulation factor activity levels or historical bleeding (eg, International Society on Thrombosis and Haemostasis Bleeding Assessment Tool [ISTH-BAT] scores) and clinical bleeding severity grade)—may not fully capture the disease burden within an individual [2,12]. Patient-reported outcomes offer a valuable tool to measure health-related quality of life (HRQoL), thereby potentially recognizing disease burden in persons with RBDs more accurately.

HRQoL has been evaluated in patients with other autosomal inherited bleeding disorders: patients with congenital platelet disorders and bleeding disorders of unknown cause (BDUC) have previously shown impaired HRQoL compared with reference populations [[13], [14], [15], [16], [17]]. However, studies focusing on RBDs are scarce [18,19]. In women with inherited bleeding disorders, heavy menstrual bleeding (HMB) has been linked to impaired HRQoL [[20], [21], [22], [23]]. As a history of HMB was reported by 80% of women in the Rare Bleeding Disorders in the Netherlands (RBiN) study, including those with mild deficiencies, this underscores the relevance of evaluating its impact on HRQoL within the RBD population specifically [24].

This study aimed to assess HRQoL in Dutch patients with RBDs enrolled in the RBiN study. Subsequently, we explored the association between disease-specific clinical characteristics and HRQoL outcomes within the RBiN population.

## 2 Methods

### 2.1 Rare Bleeding Disorders in the Netherlands study

The RBiN study was a cross-sectional, nationwide study conducted from 2017-2019 in all Dutch hemophilia treatment centers. This study included 263 patients with rare coagulation factor deficiencies (fibrinogen, factor [F]II, FV, combined FV and FVIII, FVII, FX, FXI, or FXIII deficiency) and fibrinolytic disorders (plasminogen activator inhibitor-1 deficiency, α-2-antiplasmin deficiency, or hyperfibrinolysis). All patients had been previously diagnosed based on reduced factor activity levels, abnormal fibrinolytic parameters, or through the confirmed presence of a (likely) pathogenic variant in an RBD gene [1,4]. A physician assessed all ISTH-BAT scores and category of clinical bleeding severity (grade 0 [asymptomatic], grade I, grade II, or grade III), based on historical bleeding. Grade I bleeding corresponds to provoked bleeding (following trauma and/or antithrombotic therapy), grade II corresponds to nonmajor bleeding, and grade III to major bleeding (joint, gastrointestinal, central nervous system, and umbilical cord bleeding, or intramuscular hematomas requiring hospitalization) [2]. All participants provided informed consent according to the Declaration of Helsinki. This study was approved by the Medical Research Ethical Committee Oost–Nederland and registered under the number NL61027.091.17.

### 2.2 Patient-reported measures

Patients aged ≥18 years were invited to complete comprehensive questionnaires, while those aged <18 years received a pediatric version. Information on chronic health conditions (listed in Supplementary Table S1) was not available for children. Generic questions covered demographic and clinical characteristics. After providing informed consent, patients received a personal login code for an electronic questionnaire. Reminders were sent out after 1 month if the questionnaire was not completed. Patients unable to fill out the questionnaire electronically were provided a hard copy.

Reported bleeds in the past 12 months were calculated if numerical values were reported by the patient for “Joint bleeds,” “Intramuscular hematomas,” “Bleeding due to trauma,” and/or “Other bleeds.” Patients with normal ISTH-BAT scores (<3 in children, <4 in men, or <6 in women) and a bleeding grade of 0 were considered asymptomatic [25].

Patients with grade I or II bleeding were considered to have a mild-to-moderate bleeding phenotype. A severe bleeding phenotype was defined as grade III bleeding, ≥5 reported bleeds in the past 12 months, or use of prophylaxis [2,11]. Patient-reported disease severity was determined based on a 5-point scale: “very severe,” “severe,” “rather severe,” “not severe,” and “not at all severe.” For subgroup analyses, a dichotomous division into “patient-reported nonsevere disease” and “patient-reported severe disease” was used. HMB was defined as ≥1 point scored on the ISTH-BAT item menorrhagia.

#### 2.2.1 HRQoL in adults

Patients aged ≥18 years were invited to fill out the 36-item Short Form survey (SF-36) version 2.0 and the Patient-Reported Outcome Measurement Information System, Profile 29 (PROMIS-29) version 2.0 [26,27]. The SF-36 assesses HRQoL across 8 domains: physical functioning, role limitations caused by physical health problems, bodily pain, general health, vitality, social functioning, role limitations caused by emotional problems, and mental health [28,29]. The PROMIS-29 demonstrates a significant overlap with the SF-36 and assesses 7 HRQoL domains, measured by 4-item short forms, and a separate pain intensity item, scaled from 0 to 10. These 7 domains are: physical function, anxiety, depression, fatigue, sleep disturbance, ability to participate in social roles and activities, and pain interference [27].

Scoring instructions are significantly different for both measures. For SF-36, raw scale scores are linearly converted to a 0 to 100 scale. Subscale scores were calculated if at least half of the items were completed, with a higher score indicating a better health status. For PROMIS-29, each item (excluding the pain intensity item) uses a 5-point Likert scale. Domain scores were calculated as T-scores using the Health Measures Scoring Service, resulting in a normalized T-score with a mean of 50 and a SD of 10 in the US reference population [30]. Moreover, a higher score indicates a higher degree of the construct being measured, eg, better physical function or more anxiety.

Physical and mental component summary scores (PCS-36 and MCS-36) were calculated adhering to standard scoring instructions for SF-36 [[31], [32], [33], [34], [35]]. Component summary scores (PCS-29 and MCS-29) for PROMIS-29 were calculated with Dutch scoring coefficients according to the method of Hays et al. [36,37]. Reference data from the general Dutch population are available for both SF-36 and PROMIS-29 [[38], [39], [40], [41], [42], [43]].

#### 2.2.2 HRQoL in children

The Canadian Hemophilia Outcomes–Kids Life Assessment Tool (CHO-KLAT) version 2.0 (Young et al., 2013) was used to assess HRQoL in children with RBDs. It contains 35 items based on experiences in the past 4 weeks and provides a single score ranging from 0 (worst score) to 100 (optimal score). Questions related to bleeding or treatment with factor concentrates in the past 4 weeks were not completed if no bleeding or treatment had occurred during that period. Mean scores were calculated based on available responses, as long as missing items did not exceed 50%.

### 2.3 Statistical analyses

All statistical analyses were performed using R version 4.4.1 (www.R-project.org). Categorical variables were presented as counts and percentages. Means and SDs were reported for HRQoL measures, following reporting conventions of reference populations and the SF-36 User’s Manual [33,35,[38], [39], [40], [41], [42], [43]]. PROMIS-29 scores in the RBiN population were categorized as within normal limitations or mild, moderate, or severe limitations based on Dutch reference values (Supplementary Table S2) [[39], [40], [41], [42], [43]].

#### 2.3.1 Estimating mean differences in HRQoL domains scores

Mean differences (MDs) between RBD patients and Dutch reference populations were calculated by subtracting Dutch reference means from individual patient scores. Using the individual difference from the reference population as the dependent variable, we applied a multivariable linear regression model, with sex, age, and chronic health conditions as independent variables, to adjust for these factors. To improve the robustness of our estimate, particularly given the nonnormal distribution of HRQoL scores, we repeated this analysis in 2000 resamples, a technique called bootstrapping. This approach allows for the estimation of reliable means, SDs and CIs by repeatedly drawing samples from the original data.

#### 2.3.2 Clinical subgroup analysis

A similar approach was used to compare subgroups within the RBiN population. Subgroups were based on clinical phenotype, a history of HMB, patient-reported severity, use of prophylaxis in patients with a severe phenotype, zygosity, and diagnostic rationale. For each subgroup, we estimated the adjusted mean HRQoL scores using 2000 bootstrap resamples, applying a multivariable linear regression model with sex, age, and chronic health conditions as independent variables. The MDs between subgroups were then calculated by subtracting the estimated adjusted means from each other, and MDs are reported with their 99.4% CIs.

#### 2.3.3 Minimally important differences

Beyond statistical significance, it is important to assess whether observed differences are also clinically meaningful. Minimally important differences (MIDs) refer to the smallest change in a patient-reported outcome that is noticeable or meaningful to a patient. For the SF-36 0 to 100 scales, MIDs are generally considered to range from 3 to 5 points. Therefore, we used a threshold of 5 points for each domain [28,44]. MIDs for the PROMIS-29 are domain specific and were based on previously published data in Dutch patients with hemophilia [45]. When no established MID was available, a threshold of one-half SD was used [46]. In this study, we reported only those differences that exceeded the respective MID, as these were considered clinically meaningful.

#### 2.3.4 Component summary scores

Multivariable linear regression was used to assess associations between physical and mental component summary scores (dependent variables) and age, sex, body mass index, chronic health conditions, ISTH-BAT scores, and the reported number of bleeds in the past 12 months (independent variables).

#### 2.3.5 Statistical significance thresholds

Significance thresholds (*P* ≤ .05) were adjusted for multiple testing using Bonferroni correction when appropriate. For example, statistical significance was defined as a 99.4% CI not including zero or *P* ≤ .006 in our analyses of HRQoL domains assessed using SF-36 and PROMIS-29.

## 3 Results

### 3.1 Patient characteristics

Of 206 patients with RBDs aged ≥18 years who were invited to fill out adult questionnaires, 167 (81%) completed ≥1 item of the SF-36 or PROMIS-29. A total of 40 of 57 children aged <18 years (60%) completed ≥1 item on the CHO-KLAT. Table 1 presents patient characteristics for the adult and pediatric RBiN populations. The majority of participants were female (65%) in both the adult (109/167) and pediatric (26/40) populations. A mild-to-moderate bleeding phenotype was the most common, observed in 66% (99/150) of adults and 61% (17/28) of children. In adults, 41% (65/159) were diagnosed because of bleeding symptoms and 42% (67/159) because of an affected family member. In the pediatric population, 39% (14/36) were diagnosed because of bleeding symptoms and 44% (16/36) because of an affected family member. All RBDs were represented among the adult RBiN population (Table 2).

**Table 1:** Patient characteristics for the adult and pediatric Rare Bleeding Disorders in the Netherlands population.

Characteristic	Adults (age ≥18 y) *n* = 167	Children (age <18 y) *n* = 40
**Age (y), mean (SD)**	47.8 (16.5)	10.5 (6.0)
**ISTH-BAT, median (IQR)**	10 (6-16)	5 (4-7)
**Sex**		
Female	109 (65)	26 (65)
Male	58 (35)	14 (35)
**Clinical bleeding phenotype**^a^		
Asymptomatic	5 (3)	4 (14)
Mild-to-moderate	99 (66)	17 (61)
Severe	46 (31)	7 (25)
**Prophylaxis**^b^	12 (7)	4 (10)
**Diagnostic rationale**^c^		
Bleeding symptoms	65 (41)	14 (39)
Abnormal coagulation tests	27 (17)	6 (17)
Affected family member	67 (42)	16 (44)
**Concomitant chronic health conditions**^d^		
None	76 (46)	
1	53 (32)	
>1	37 (22)	
**Body mass index**^e^		
<25 kg/m^2^	72 (45)	
25-30 kg/m^2^	55 (39)	
≥30 kg/m^2^	21 (16)	
**Genotype**^f^		
Biallelic	37 (29)	
Heterozygous, autosomal recessive	63 (50)	
Heterozygous, autosomal dominant	8 (6)	
Heterozygous, 2 variants phase unknown	5 (4)	
No variant	13 (10)	
**Education**^g^		
Primary	37 (26)	
Secondary	46 (32)	
Tertiary	60 (42)	

Values are *n* (%) unless otherwise stated.

ISTH-BAT, International Society on Thrombosis and Haemostasis Bleeding Assessment Tool; RBiN, Rare Bleeding Disorders in the Netherlands.

a Information on clinical bleeding phenotype missing in 17 adult participants (10%) and 12 children (30%).

b Information on prophylaxis missing in 18 adult participants (11%).

c Information about diagnostic rationale missing in 8 adult participants (5%) and 4 children (10%).

d Information about chronic health conditions missing in 1 adult participant (1%). Not available for children.

e Body mass index missing in 7 adult participants (4%).

f No genotyping performed in 41 adult participants (25%) and not standardly performed in children in the RBiN study.

g Information about education missing in 24 adult patients (14%). Not available for children.

**Table 2:** An overview of rare bleeding disorders in the adult and pediatric Rare Bleeding Disorders in the Netherlands population.

Rare bleeding disorder	Adult RBiN population	Pediatric RBiN population
	*N* (%)	*N* (%)
Fibrinogen disorder	26 (16)	5 (13)
FII deficiency	10 (6)	1 (2,5)
Combined FV and FVIII deficiency	2 (1)	1 (2,5)
FV Amsterdam	2 (1)	
FV deficiency	18 (11)	3 (7,5)
FVII deficiency	33 (20)	14 (35)
FX deficiency	5 (3)	
FXI deficiency	23 (14)	9 (22,5)
FXIII deficiency	6 (4)	4 (10)
Hyperfibrinolysis	14 (8)	
PAI-1 deficiency	11 (7)	
A2AP deficiency	17 (10)	3 (7,5)
Total	167 (100)	40 (100)

A2AP, α-2 antiplasmin; F, factor; PAI-1, plasminogen inhibitor activator 1; RBiN, Rare Bleeding Disorders in the Netherlands.

### 3.2 HRQoL in persons with RBDs compared with the general population

**Figure 1:**
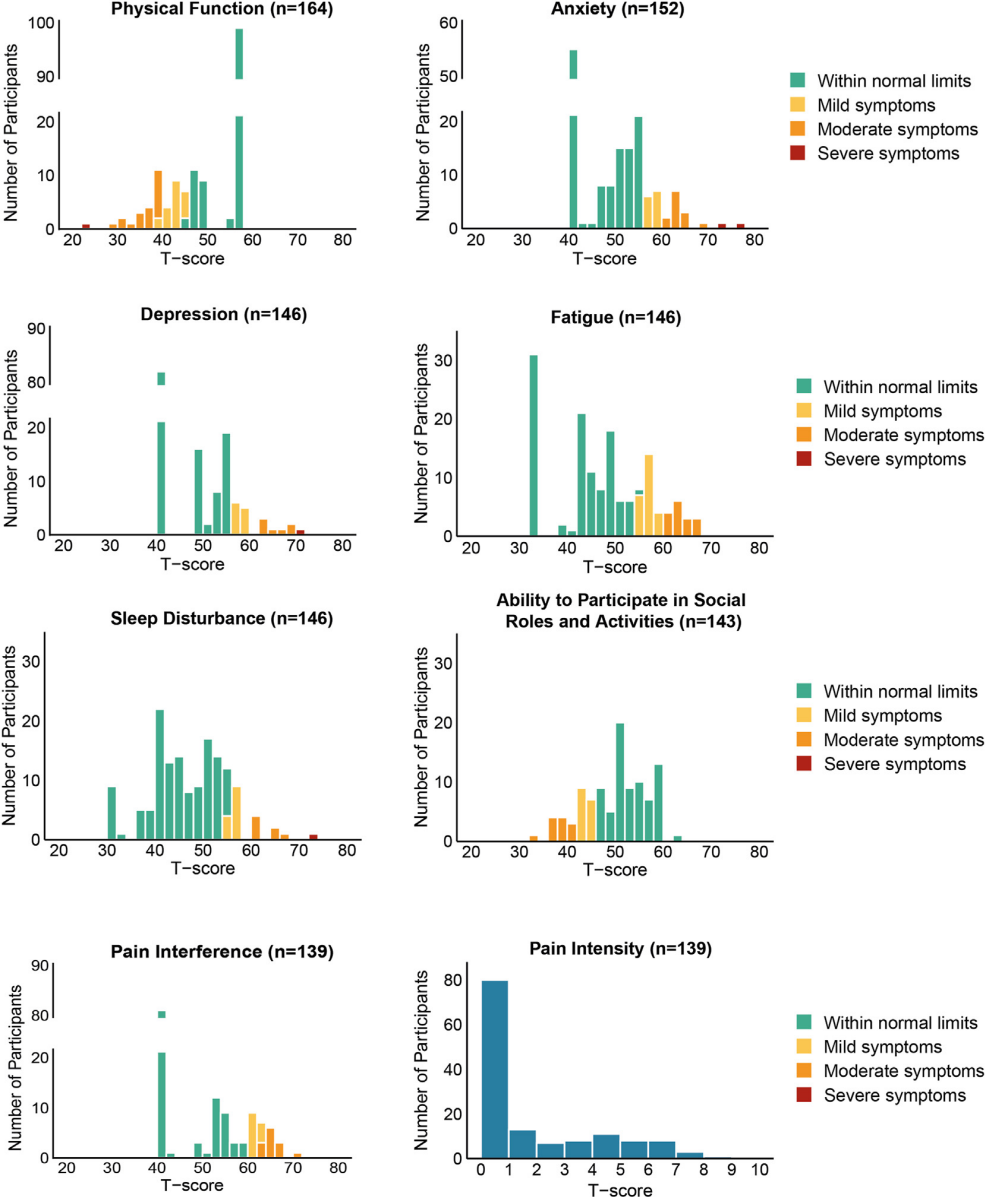
Distribution of T-scores for PROMIS-29 domains and the level of pain intensity within the RBiN population. Domain specific cutoff values that classify scores as within normal limits, or indicate mild, moderate, and severe symptoms were based on Dutch reference populations and are shown in Supplementary Table S2 [[38], [39], [40], [41], [42], [43]]. PROMIS-29, Patient-reported Measurement Information System, Profile 29; RBiN, Rare Bleeding Disorders in the Netherlands.

Of 144 adults who started the SF-36 questionnaire, 115 (80%) completed it. Compared to the Dutch reference, no statistically significant differences were observed in HRQoL domains (Table 3) [38]. The PROMIS-29 was completed by 139 (85%) of the 164 adults that started it. Figure 1 presents RBiN participants’ T-scores across 7 PROMIS-29 domains and the pain intensity item. Compared with Dutch reference populations, patients with RBDs reported less sleep disturbance, a greater ability to participate in social roles/activities, and less pain interference and intensity (Table 4). Demographics for reference populations for SF-36 and PROMIS-29 domains are summarized in Supplementary Table S3 [[38], [39], [40], [41], [42], [43]].

**Table 3:** Estimated means and mean differences across 36-item Short Form survey domains between adult patients from the Rare Bleeding Disorders in the Netherlands study and the Dutch reference population [1].

SF-36 domain	MID	RBiN		Dutch reference	MD^b^	99.4% CI
		*n*	Mean^a^ (SD)	Mean^a^ (SD)		
Physical functioning	≥5	136	88.1 (34.5)	83.9 (20.7)	**5.7**	**−4.6, 14.8**
Role limitation due to physical health	≥5	137	75.9 (23.8)	77.0 (37.0)	0.6	−15.8, 17.8
Bodily pain	≥5	130	77.5 (26.8)	75.1 (24.9)	2.6	−7.4, 14.3
General health	≥5	115	66.4 (22.2)	71.4 (20.6)	**−6.1**	**−14.7, 3.6**
Vitality	≥5	123	61.3 (24.5)	68.2 (20.7)	−2.2	−13.2, 9.3
Social functioning	≥5	131	82.0 (32.5)	84.2 (20.8)	0.2	−9.9, 10
Role limitations due to emotional problems	≥5	133	87.4 (30.4)	82.2 (37.0)	4.6	−6.9, 15.1
Mental health	≥5	123	74.9 (28.1)	76.5 (16.6)	−1.5	−7.9, 5.5

MDs did not reach statistical significance (*P* ≤ .006). Differences exceeding MIDs are highlighted in **bold**. Lower scores indicate worse outcomes.

MD, mean difference; MID, minimally important difference; RBiN, Rare Bleeding Disorders in the Netherlands; SF-36, 36-item Short Form survey.

a Adjusted for age and sex.

b Adjusted for age, sex, and chronic health conditions.

**Table 4:** Estimated means and mean differences across PROMIS-29 domains and the pain intensity item between adults patients from the Rare Bleeding Disorders in the Netherlands study and the Dutch reference [[2], [3], [4], [5], [6]].

PROMIS-29 domain	MID	RBiN		Dutch reference	MD^b^	99.4% CI
		*n*	Mean^a^ (SD)	Mean^a^ (SD)		
Physical function	≥2	164	51.2 (7.9)	49.8 (10.8)	1	−1.1, 2.8
Anxiety	≥−2.3	152	49.7 (8.5)	49.9 (10.1)	−0.4	−2.4, 1.6
Depression	≥−3.0	146	47.2 (7.8)	49.6 (10.0)	−2.2∗	−4.0, −0.3
Fatigue	≥−2	146	47.5 (9.5)	49.1 (10.8)	−1.3	−3.5, 0.6
Sleep disturbance	≥−1	146	47.4 (7.8)	49.7 (9.4)	**−2.1∗**	**−4.2, −0.2**
Ability to participate in social roles/activities	≥1	143	54.1 (8.5)	50.6 (9.5)	**3.5∗**	**1.4, 5.6**
Pain interference	≥−2.0	139	48.6 (9.0)	54.9 (8.6)	**−6.0∗**	**−8.0, −3.9**
Pain intensity	≥1	139	2.1 (2.5)	3.1 (2.7)	**−1.0∗**	**−1.6, −0.4**

Differences exceeding MIDs are highlighted in **bold**. An asterisk (∗) indicates a significant difference (*P* ≤ .006). A higher score indicates more of the construct.

MD, mean difference; MID, minimally important difference; PROMIS-29, Patient-Reported Measurement Information System, Profile 29; RBiN, Rare Bleeding Disorders in the Netherlands.

a Unadjusted.

b Adjusted for age, sex, and chronic health conditions.

Patients who completed either the SF-36 or the PROMIS-29 questionnaire did not differ significantly from those who did not in terms of age, sex, number chronic health conditions, body mass index, education level, ISTH-BAT scores, or clinical phenotype (data not shown).

### 3.3 Subgroup analyses across HRQoL domains

Analyses of subgroups within the RBiN population based on clinical bleeding phenotype, history of HMB, and patient-reported severity in SF-36 and PROMIS-29 are summarized in Tables 5 and Table 6. The number of respondents varied per domain, as 80% to 85% of participants completed the questionnaires.

**Table 5:** Estimated mean differences across 36-item Short Form survey domains and summary component scores across patient subgroups.

SF-36 domain	MID	Severe (*n* = 41-36) vs mild-to-moderate bleeding phenotype (*n* = 85-68)	Women with a history of HMB (*n* = 69-57) vs women without (*n* = 14-12)	Patient-reported severe disease (*n* = 46-37) vs nonsevere (*n* = 86-74)
		MD^a^ (99.4% CI)	MD^b^ (99.4% CI)	MD^a^ (99.4% CI)
Physical functioning	**≥5**	**−9.5 (−21.3, 2.0)**	**−15.8∗ (−29.9, −6.7)**	**−6.9 (−18.4, 5.0)**
Role limitation due to physical health	**≥5**	**−10.5 (−31.5, 10.7)**	**−24.1 (−48.1, 6.2)**	**−12.1 (−31.8, 6.9)**
Bodily pain	**≥5**	**−12.9 (−27.4, 3.1)**	**−21.3∗ (−34.5, −8.0)**	**−11.6 (−25.1, 3.0)**
General health	**≥5**	**−12.4 (−24.0, 0.8)**	**−14 (−30.8, 0.7)**	**−12.0 (−25.2, 0.9)**
Vitality	**≥5**	−3.5 (−15.1, 7.1)	−1.4 (−21.2, 18.7)	**−7.2 (−16.9, 2.7)**
Social functioning	**≥5**	**−9.6 (−21.2, 3.2)**	**−7.5 (−21.7, 11.4)**	**−8.5 (−20.8, 3.3)**
Role limitations due to emotional problems	**≥5**	0.1 (−15.7, 19.3)	**−9.5 (−24.5, 10.6)**	**−7.1 (−20.9, 5.8)**
Mental health	**≥5**	1.5 (−10.3, 12.2)	−0.6 (−19.3, 23.5)	**−5.0 (−15.0, 4.9)**

Component summary scores				
Physical health (PCS-36)	≥2	**−6.5∗ (−11.7, −1.2)**	**−9.0∗ (−15.5, −2.6)**	**−5.7 (−11.6, 0.4)**
Mental health (MCS-36)	≥3	0,6 (−5.2, 5.9)	−1.4 (−8.2, 8.8)	−2.2 (−7.1, 3.2)

Differences exceeding MIDs are highlighted in bold. An asterisk (∗) indicates a significant difference (*P* ≤ .006). Lower scores indicate worse outcomes.

HMB, heavy menstrual bleeding; MCS, mental component summary score; MD, mean difference; MID, minimally important difference; PCS, physical component summary score; RBD, Rare Bleeding Disorder; SF-36, 36-item Short Form survey.

a Adjusted for age, sex, and chronic health conditions.

b Adjusted for age and chronic health conditions.

**Table 6:** Estimated mean differences across PROMIS-29 domains, the pain intensity item, and summary component scores across patient subgroups.

PROMIS-29 domain	MID	Severe (*n* = 46-39) vs mild-to-moderate bleeding phenotype (*n* = 96-83)	Women with a history of HMB (*n* = 77-67) vs women without (*n* = 15-12)	Patient-reported severe disease (*n* = 53-50) vs nonsevere (*n* = 94-81)
		MD^a^ (99.4% CI)	MD^b^ (99.4% CI)	MD^a^ (99.4% CI)
Physical function	**≥−2**	**−2.7 (−7.0, 1.7)**	**−3.9 (−8.1, 0.8)**	**−3.3 (−7.1, 0.8)**
Anxiety	**≥2.3**	−1.4 (−6.0, 3.4)	−0.2 (−9.3, 8.2)	**3.0 (−0.9, 7.1)**
Depression	**≥3.0**	−1.3 (−5.7, 3.3)	−0.6 (−8.9, 6.7)	1.1 (−2.5, 5.1)
Fatigue	**≥2**	−1.6 (−6.7, 4.2)	**3.2 (−5.6, 12.1)**	**4.2 (−0.8, 9.7)**
Sleep disturbance	**≥1**	**1.5 (−3.1, 6.3)**	**2.0 (−5.8, 8.9)**	**2.9 (−1.3, 6.9)**
Ability to participate in social roles/activities	**≥−1**	**−2.7 (−6.9, 2.2)**	**−5.3 (−11.8, 1.5)**	**−4.2 (−8.6, 0.4)**
Pain interference	**≥2.0**	**3.4 (−1.3, 8.4)**	**6.4∗ (0.5, 12.7)**	**5.2∗ (0.7, 9.8)**
Pain intensity	**≥1**	**1.0 (−0.3, 2.3)**	**1.8∗ (0.3, 3.5)**	0.9 (−0.4, 2.2)

Component summary scores				
Physical health (PCS-29)	≥6.2	−4.6 (−11.4, 2.7)	−6.1 (−13.5, 4.1)	−5.9∗ (−12.4, 0.9)
Mental health (MCS-29)	≥4.7	−0.2 (−5.7, 5.1)	−3.9 (−12.6, 7.1)	**−4.9∗ (−9.9, −0.4)**

Differences exceeding MIDs are highlighted in bold. An asterisk (∗) indicates a significant difference (*P* ≤ .006). A higher score indicates more of the construct.

HMB, heavy menstrual bleeding; MCS, mental component summary score; MD, mean difference; MID, minimally important difference; RBD, rare bleeding disorder, PCS, physical component summary score; PROMIS-29, Patient-Reported Measurement Information System, Profile 29.

a Adjusted for age, sex, and chronic health conditions.

b Adjusted for age and chronic health conditions.

Compared with patients with a mild-to-moderate bleeding phenotype, patients with a severe bleeding phenotype reported significantly worse physical health (MD: −6.5). As only 5 patients with an asymptomatic phenotype were included, it was decided that this sample size was too small to conduct meaningful statistical analyses. However, estimated mean (SD) for patients with an asymptomatic, mild-to-moderate, and severe phenotype are provided in Supplementary Table S4 and S5.

Those with patient-reported severe disease scored significantly worse on PROMIS-29 domain “pain interference” (MD: 5.2) and both physical (MD: −5.9) and mental health (MD: −4.9) compared with those with patient-reported nonsevere disease (Table 6). While not statistically significant, patients with reported severe disease scored worse on psychosocial domains such as vitality, role limitations because of emotional problems, mental health (SF-36), anxiety, and fatigue (PROMIS-29) compared with those with patient-reported nonsevere disease. Among patients with a severe phenotype, 41% (18/43) reported their disease as “not at all severe” or “not severe.” Conversely, 32% of patients with a mild-to-moderate phenotype (28/87) reported their disease as “rather severe” (*n* = 17), “severe” (*n* = 10), or “very severe” (*n* = 1) (see Supplementary Table S6).

Of participants included in this substudy, 77 women had a history of HMB, and 15 had no history of HMB. Nearly half (45%, 41/77) of these women were premenopausal, with 85% (35/41) reporting HMB. Women with a history of HMB had significantly lower HRQoL scores measured by SF-36 compared with those without a history of HMB for physical functioning (MD: −15.8), bodily pain (MD: −21.3), and overall physical health (MD: −8.4) (Table 5). Moreover, they reported worse pain interference (MD: 6.4) and pain intensity (MD: 1.8) on PROMIS-29 (Table 6).

Supplementary Tables S7 and S8 summarize results of subgroup analyses within the RBiN based on the use of prophylaxis in patients with a severe bleeding phenotype, zygosity, and diagnostic rationale. Patients with a severe phenotype using prophylaxis reported better mental health measured by MCS-29 than did those without prophylaxis (MD: 9.1). Moreover, patients diagnosed because of bleeding symptoms reported more role limitations due to emotional problems compared with patients diagnosed because of an affected family member (MD: −13.9). All other differences between subgroups were not statistically significant.

### 3.4 Summary component scores

ISTH-BAT scores were significantly associated with lower physical health measured by both SF-36 and PROMIS-29 component summary scores (Table 7). Age was associated with lower PCS-29. None of the other independent variables were associated with impaired physical health. For MCS-29, presence of >1 chronic health condition was significantly associated with worse mental health, but not for MCS-36. None of the other studied characteristics were associated with impaired mental health.

**Table 7:** Results of multiple linear regression analyzing the association between clinical characteristics and physical component summary score and mental health component summary score, measured by component summary scores based on 36-item Short Form survey and Patient-Reported Measurement Information System, Profile 29 results.

	SF-36				PROMIS-29			
	PCS-36		MCS-36		PCS-29		MCS-29	
*n*	111		111		139		139	
Mean (SD)	49.6 (10.0)		51.2 (8.1)		52.0 (12.4)		53.9 (9.4)	

Participants included in model, *n* (%)	91		91		109		109	
Characteristic	β	99% CI	β	99% CI	β	99% CI	β	99% CI
Age	−0.1	−0.3, 0.1	0.1	−0.1, 0.2	−0.3∗	−0.5, −0.1	0.0	−0.1, 0.2
Male sex	4.0	−1.1, 8.7	2.3	−3.2, 7.2	1.7	−3.6, 6.8	3.9	−0.4, 8.0
BMI	0.0	−0.7, 0.7	−0.3	−0.9, 0.3	−0.6	−1.3, 0.2	−0.3	−0.9, 0.4
1 chronic health condition	1.8	−5.3, 9.6	1.7	−5.4, 8.8	−1.6	−10.7, 7.0	3.5	−3.5, 9.6
>1 chronic health conditions	1.8	−6.4, 10.9	4.2	−1.9, 10.2	0.4	−8.0, 8.6	7.3∗	0.7, 13.8
ISTH-BAT	−0.5∗	−0.8, −0.1	0.1	−0.3, 0.4	−0.5∗	−0.9, −0.1	−0.1	−0.4, 0.2
No of bleeds in past 12 mo	−0.3	−1.0, 0.0	−0.1	−1.5, 0.5	−0.5	−1.0, 0.2	−0.2	−1.0, 0.3

An asterisk (∗) indicates a significant difference (*P* ≤ .008).

β, regression coefficient; BMI, body mass index; ISTH-BAT, International Society on Thrombosis and Haemostasis Bleeding Assessment Tool; MCS, mental component summary score; PCS, physical component summary score; PROMIS-29, Patient-Reported Measurement Information System, Profile 29; SF-36, 36-item Short Form survey.

### 3.5 CHO-KLAT

**Figure 2:**
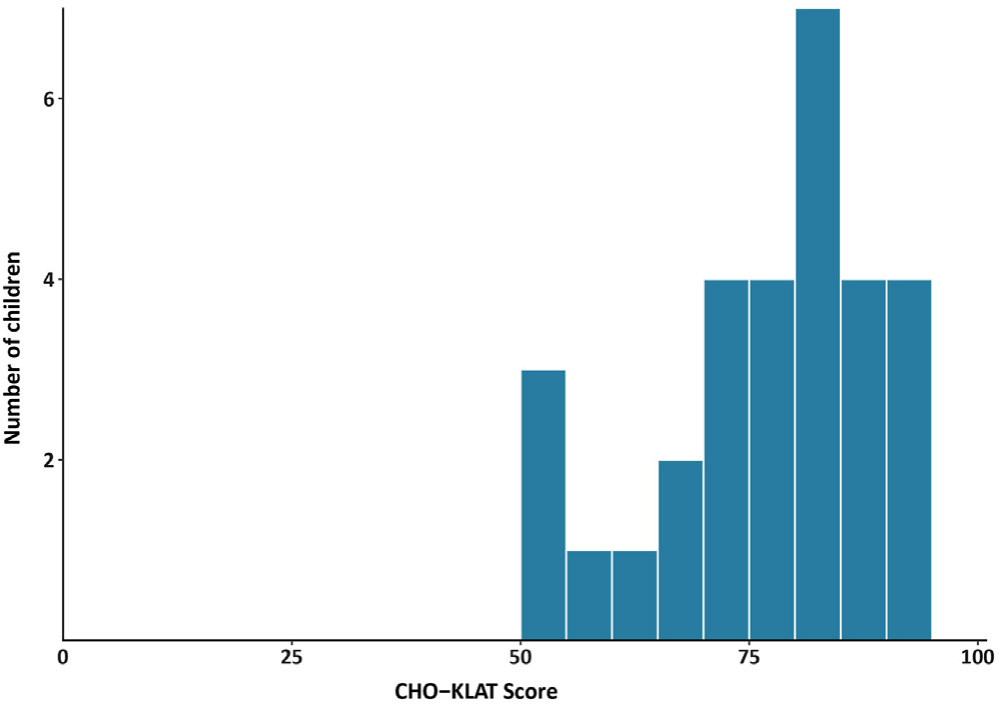
Distribution of Canadian Hemophilia Outcomes–Kids Life Assessment Tool (CHO-KLAT) scores in the pediatric Rare Bleeding Disorders in the Netherlands (RBiN) population.

A total of 29 of 40 children (73%) reached a response rate >50% in the CHO-KLAT questionnaire. Results are summarized in Table 8, and the distribution is shown in Figure 2. The overall mean score for these 29 children was 76.9 (SD: 11.4). Overall, girls scored lower than boys (MD: −6.0; 95% CI: –13.2, 1.9), and children with self-reported severe disease scored worse than children reporting nonsevere disease (MD: −8.1; 95% CI: –23.3, 3.9). However, these differences were not statistically significant.

**Table 8:** The Canadian Hemophilia Outcomes–Kids Life Assessment Tool scores of the pediatric Rare Bleeding Disorders in the Netherlands population, stratified by sex, patient (or caregiver)-reported disease severity, and clinical bleeding phenotype.

Characteristic	*n*	Mean (SD)	95% CI
Total	29	76.9 (11.4)	72.9, 80.8
Sex			
Female	21	75.2 (12.1)	69.9, 80.1
Male	8	81.2 (7.3)	75.2, 86.0
Patient/caregiver-reported disease severity^a^			
Severe	4	73.0 (12.1)	58.9, 83.5
Nonsevere	15	81.2 (8.5)	76.4, 85.4
Clinical bleeding phenotype^b^			
Asymptomatic	3	74.0 (22.8)	54.2, 85.7
Mild-to-moderate	12	77.5 (8.9)	71.7, 82.4
Severe	7	79.9 (11.8)	69.6, 87.8

CHO-KLAT, Canadian Hemophilia Outcomes–Kids Life Assessment Tool; RBiN, Rare Bleeding Disorders in the Netherlands.

a Missing in 10 children (34%).

b Missing in 7 children (26%).

## 4 Discussion

To our knowledge, this is the first study to assess HRQoL in a nationwide cohort of adult and pediatric patients with RBDs. Overall, the HRQoL in patients with RBDs was at least comparable with the Dutch reference populations. However, we identified disease-specific characteristics that appear to contribute to HRQoL on an individual level. Within the RBiN population, severe bleeding phenotype, history of HMB, and patient-reported severe disease emerged as significant factors associated with impaired HRQoL. Additionally, higher ISTH-BAT scores were negatively associated with physical health.

### 4.1 HRQoL in RBDs

Overall, HRQoL scores in the RBiN cohort were comparable to, or perhaps even slightly better than, those in Dutch reference populations. This relatively normal HRQoL in patients living with RBDs may partly reflect high health literacy and an effect of regular monitoring in hemophilia treatment centers, whereby even mildly affected patients might benefit from structured care. Additionally, a high proportion of individuals in the RBiN cohort had received tertiary education (42%), compared with 25% in the SF-36 and approximately 30% in the PROMIS-29 reference populations. The RBiN population also reported a lower prevalence of multiple chronic health conditions (22%) compared with the SF-36 reference group (42%) [38]. As HRQoL tends to be the highest in individuals with higher education, and the presence of chronic health conditions may negatively impact HRQoL, these differences could be relevant and therefore important to mention [47]. Another possible explanation for the relative normal HRQoL in the RBiN cohort is the large variability in clinical severity among patients with RBDs, which may have impaired the possibility to detect significant differences at a group level.

Although we did not observe reduced HRQoL in patients with RBDs compared with reference populations, decreased HRQoL has been previously reported in patients with BDUC and congenital platelet disorders. This may be partly explained by the absence of patients diagnosed solely through family screening in the BDUC and Trombocytopathy in the Netherlands cohorts, resulting in a relative overrepresentation of individuals with more severe disease in those cohorts compared with our study population [16,17]. Moreover, our study applied a more stringent approach by correcting conservatively for multiple testing and reporting only those differences exceeding the MID threshold.

### 4.2 HMB and HRQoL

Female participants who reported HMB had notably worse scores on pain-related items compared to those without a history of HMB. Possibly, presence of HMB-associated menstrual pain may contribute to reduced HRQoL in women with RBDs. However, 45% of women with a history of HMB in the RBiN were premenopausal, indicating that the observed differences in HRQoL cannot be solely attributed to ongoing menstrual bleeding [24].

Physical health measured by the SF-36 domain “physical functioning” and PCS-36 was also significantly worse. While it has been hypothesized that low HRQoL in women with inherited bleeding disorders may be partially attributed to iron deficiency anemia, hemoglobin levels were not significantly different in both groups, even after selecting only premenopausal women (data not shown). Given that HMB is inherently a patient-reported symptom, its presence—or history—may reflect a broader disease burden, as 41% (28/69) of women with HMB reported severe disease. This accentuates the importance of addressing HMB in women with inherited bleeding disorders, and RBD specifically, as improved management of women with HMB may enhance HRQoL in premenopausal women [20,21,48].

### 4.3 Other disease-specific patient characteristics associated with HRQoL

One of the primary goals of this study was to identify relevant disease-specific patient characteristics associated with HRQoL outcomes in persons with RBDs. We hypothesized that a severe bleeding phenotype would be associated with worse HRQoL, which was supported by lower PCS-36 scores in patients with a severe phenotype than in those with a mild-to-moderate phenotype. Worse mental health in patients with reported severe disease may suggest that patient-reported severity captures the psychosocial burden in patients with RBDs more effectively than clinical bleeding phenotype. Notably, patients with a severe bleeding phenotype who did not receive prophylaxis had significantly worse mental health scores than those receiving prophylaxis.

Our findings underscore the importance of integrating patient-reported severity into disease burden assessments in patients with RBDs to complement established classifications. Further research is needed to explore why some patients with RBDs perceive their disease as severe and to define an optimal cutoff for defining patient-reported severity. Additionally, the potential benefits of prophylaxis in this heterogeneous population should be evaluated and weighed against costs and treatment burden. In this context, nonconventional prophylactic approaches, such as periodic treatment around menstruation, may offer a more targeted and less burdensome approach for certain patients.

### 4.4 HRQoL in children with RBDs

HRQoL in the pediatric RBiN population measured by the CHO-KLAT version 2.0 (mean: 76.9 [SD: 11.4]) was almost equal to reported mean scores (mean: 77.0 [SD: 11.2]) in boys with hemophilia in a validation study across 5 European countries [49]. Being bothered by the disease was the sole factor influencing HRQoL in the only study investigating HRQoL in children with RBDs [18]. While we observed the same trend in patient-reported severe disease compared with in nonsevere disease, these differences were not significant. The applicability of the CHO-KLAT in our population remains uncertain, as it has only been validated in boys with hemophilia, with 3 of its 35 questions specifically addressing factor concentrate treatment. Additionally, generic pediatric HRQoL questionnaires were not administered in the RBiN study, which limits broader comparisons.

### 4.5 Study limitations

The main limitation of this retrospective study is selection bias. Nonresponse bias may have led to the inclusion of patients with a higher socioeconomic status. Given the small number of patients in certain subgroups, inherent to research on rare disorders, results of our study should be interpreted accordingly. Further research on HRQoL in other populations with RBDs is necessary to confirm our findings. As ethnicity was not assessed in the RBiN study, its potential influence on HRQoL in patients with RBDs remains unknown. Additionally, missing data occurred due to drop-down menu errors within the computer-assisted answering system in 4 patients who reported limitations in daily activities and were unable to specify which activities were affected.

HRQoL was assessed at a single time point. Given the observed impact of HMB on HRQoL in women with RBDs, it would have been valuable to collect information on the timing of the questionnaire completion within the menstrual cycle, as scores may be worse during menstruation for females experiencing significant symptoms [22,23].

Finally, the measures used to quantify HRQoL have certain limitations. The 4-item short form structure of the PROMIS-29 limits reliability for the domain “sleep disturbance,” rendering it unsuitable for clinical practice [27,42]. Computer adaptive testing (CAT) has been shown to outperform short forms, but PROMIS CATs were not administered in this study [40,43]. However, scores from short forms are highly correlated with those produced by CATs. While the used measures have not been specifically validated in persons with RBDs, PROMIS-29 has been validated in the Dutch population with and without chronic diseases and in patients with hemophilia, and PROMIS CATs were recently validated in persons with autosomal inherited bleeding disorders [37,45,50]. Moreover, the SF-36 is the most widely used generic instrument for measuring HRQoL and has shown good consistency, reliability, and validity in patients with inherited bleeding disorders. Nonetheless, as Dutch SF-36 reference scores are based on questionnaires administered in 1996, it is uncertain to what extent they reflect current HRQoL [38].

## 5 Conclusions

We assessed HRQoL in a large, national cohort of adult and pediatric patients with RBDs and demonstrated that overall HRQoL in the RBiN population is comparable to that of Dutch reference populations. Disease-specific factors associated with worse HRQoL included a severe clinical bleeding phenotype, patient-reported severe disease, and a history of HMB. Additionally, ISTH-BAT scores correlated with lower physical health. Incorporating patient-reported severity and HMB history as key outcomes in clinical practice may improve the assessment of current disease burden among patients with RBD and could serve as valuable endpoints in future studies in patients with inherited bleeding disorders.
