# Supplementary material for: Health-related quality of life in rare bleeding disorders: results from the Rare Bleeding Disorders in the Netherlands study
Source: Res Pract Thromb Haemost. 2025 Jun 27;9(5):102961. doi: 10.1016/j.rpth.2025.102961 (PMC12304960; doi:10.1016/j.rpth.2025.102961)
Supplement: Supplementary Material [file mmc1.docx]

**Supplementary information**

**Health-related quality of life in rare bleeding disorders: results from the Rare Bleeding Disorders in the Netherlands study**

*Sterre P.E. Willems*^1,2^*, Marjon H. Cnossen^3^, Nick van Es^4,5^, Paul L. den Exter^6^, Ilmar C. Kruis^7^, Karina Meijer^8^, Laurens Nieuwenhuizen*^9,2^*, Joline L. Saes*^10^*, Nicole M.A. Blijlevens*^1^*, Waander L. van Heerde^2, 11^, Saskia E.M. Schols*^1,2^

^1^Department of Hematology, Radboud university medical center, Nijmegen, The Netherlands

^2^Hemophilia Treatment Center, Nijmegen – Eindhoven – Maastricht, The Netherlands

*^3^*Department of Pediatric Hematology and Oncology, Erasmus MC Sophia Children ’s Hospital, University Medical Center Rotterdam, Rotterdam, The Netherlands

^4^Amsterdam UMC location University of Amsterdam, Department of Vascular Medicine,

Meibergdreef 9, Amsterdam, Netherlands

^5^Amsterdam Cardiovascular Sciences, Pulmonary Hypertension & Thrombosis, Amsterdam, The Netherlands

^6^Department of Medicine - Thrombosis and Hemostasis, Leiden University Medical Center, Leiden, The Netherlands

^7^Netherlands Hemophilia Society, Nijkerk, The Netherlands,

*^8^*Department of Hematology, University Medical Center Groningen, Groningen, The Netherlands

^9^Department of Hematology, Máxima Medical Center Eindhoven, Eindhoven, The Netherlands

^10^Center for Benign Haematology, Thrombosis and Haemostasis, van Creveldkliniek, University Medical Center Utrecht and University Utrecht, Utrecht, The Netherlands

*^11^*Enzyre BV, Novio Tech Campus, Nijmegen, The Netherlands

Corresponding author: Saskia E.M. Schols

Department of Hematology, Radboud university medical center

PO Box 9101, 6500 HB Nijmegen, the Netherlands

Tel. +31 24 361 8823

Fax. +31 24 363 1563

[Saskia.Schols@radboudumc.nl](mailto:Saskia.Schols@radboudumc.nl)

Contents:

- Supplemental Table S1, page 2
- Supplemental Table S2, page 2
- Supplemental Table S3, page 3
- Supplemental Table S4 and S5, page 4
- Supplemental Table S6, page 5
- Supplemental Table S7 and S8, page 6

Supplemental Table S1:  **List of chronic health conditions adapted from a standard list of ten chronic conditions listed by the Dutch Central Bureau of Statistics, with the addition of severe renal disease, liver disease, and mental disorders.[1]**

1. Asthma or chronic bronchitis
2. Heart defect
3. Stroke
4. High blood pressure
5. Gastrointestinal disorders
6. Diabetes
7. Back disorder
8. Rheumatic/joint disorders
9. Migraine
10. Cancer
11. Severe renal disease
12. Liver disease or cirrhosis
13. Mental disorders

Supplemental Table S2: **Cut-off values based on Dutch reference populations for symptoms within normal limits, mild, moderate, and severe symptoms per PROMIS-29 domain.[2-6]**

|  | Within normal limits | Mild symptoms | Moderate symptoms | | | Severe symptoms | |
| --- | --- | --- | --- | --- | --- | --- | --- |
| Physical function | >45 | 39-45 | 28-39 | | | <28 | |
| Anxiety | <56 | 56-60 | 61-70 | | | >70 | |
| Depression | <56 | 56-60 | 61-70 | | | >70 | |
| Fatigue | <55 | 55-59 | 60-70 | | | >70 | |
| Sleep disturbance | <55 | 55-59 | 60-69 | | | >69 | |
| Ability to participate in social roles and activities | >46 | 41-46 | 32-41 | | | <32 | |
| Pain interference | <59 | 59-63 | 63-72 | | | >72 | |
|  |  | | |  |  | |  |

Supplemental Table S3: **Demographics of Dutch reference populations.** As Dutch reference values for PROMIS item banks were obtained in several populations, demographics are displayed for the respective items.

|  |  | SF-36  reference population[7] | PROMIS reference populations | | | | | | |
| --- | --- | --- | --- | --- | --- | --- | --- | --- | --- |
|  |  |  | **Anxiety and depression[2]** | **Global health[3]** | **Fatigue[4]** | **Physial function[5]** | **Pain interference and behavior[5]** | **Ability to participate and satisfaction with participation[5]** | **Sleep[6]** |
|  |  | n=1742 | n=1002 | n=4370 | n=1006 | n=1310 | n=1052 | n=1002 | n=1006 |
| Age, y, mean (SD) | | 47.5 (18) | 49 (17) | 51 (17) | 52 (17) | 51 (17) | 51 (17) | 51 (17) | 51 (17) |
| Sex, n (%) | |  |  |  |  |  |  |  |  |
|  | Female | 44% | 52% | 53% | 53% | 53% | 53% | 52% | 51% |
|  | Male | 56% | 48% | 47% | 47% | 47% | 47% | 48% | 49% |
| Education, n (%) | |  |  |  |  |  |  |  |  |
|  | Primary/low | 47% | 32% | 29% | 28% | 31% | 28% | 30% | 28% |
|  | Secundary/Middle | 28% | 40% | 41% | 41% | 40% | 40% | 40% | 41% |
|  | Tertiary/High | 25% | 28% | 30% | 31% | 29% | 32% | 30% | 31% |
| Ethniciy | |  |  |  |  |  |  |  |  |
|  | Native | 82% | 80% | 78% | 79% | 79% | 77% | 79% | 79% |
|  | 1^st^/2^nd^ generation western immigrant | 8% | 13% | 12% | 11% | 12% | 12% | 10% | 11% |
|  | 1^st^ /2^nd^ generation non-western immigrant | 5% | 8% | 10% | 10% | 9% | 11% | 11% | 10% |
|  | Unknown | 5% | - | - | - | - | - | - | - |
| Chronic health conditions*, n (%) | | |  |  |  |  |  |  |  |
|  | None | 28% | - | - | - | - | - | - | - |
|  | 1 | 30% | - | - | - | - | - | - | - |
|  | >1 | 42% | - | - | - | - | - | - | - |

SF-36: 36-item Short Form survey; PROMIS: Patient-reported Measurement Information System

Supplemental Table S4: **Estimated mean domain scores across SF-36 domains, and summary component scores across different bleeding phenotypes.** Note: because the means (SD) were estimated by re-running the bootstrapping procedure, small differences may be observed compared to the mean differences reported in Tables 5 and 6.

|  | Asymptomatic | | | | | Mild-to-moderate | | | | | Severe | | | | | | |
| --- | --- | --- | --- | --- | --- | --- | --- | --- | --- | --- | --- | --- | --- | --- | --- | --- | --- |
| SF-36 domain | **n** | **Mean^a,b^ (SD)** | | | | **n** | | **Mean^a^ (SD)** | | | **n** | | **Mean^a^ (SD)** | | | | |
| Physical Functioning | 5 | 95.0 (19.7) | | 84 | | | 93.0 (19.2) | | | | | 38 | | 83.7 (22.8) | |  |  |
| Role limitation due to physical health | 5 | 89.2 (35.1) | | 82 | | | 81.2 (38.9) | | | | | 41 | | 70.6 (37.8) | |  |  |
| Bodily Pain | 4 | 84.9 (23.9) | | 78 | | | 82.7 (26.5) | | | | | 39 | | 69.6 (26.9) | |  |  |
| General Health | 4 | 75.2 (20.9) | | 68 | | | 69.5 (22.3) | | | | | 36 | | 57.2 (20.4) | |  |  |
| Vitality | 4 | 68.5 (16.8) | | 72 | | | 66.7 (19.5) | | | | | 38 | | 63.2 (19.1) | |  |  |
| Social Functioning | 5 | 90.4 (17.9) | | 78 | | | 88.2 (20.3) | | | | | 39 | | 78.4 (21.9) | |  |  |
| Role limitations due to emotional problems | 5 | 91.4 (27.5) | | 80 | | | 87.0 (37.6) | | | | | 39 | | 87.0 (26.2) | |  |  |
| Mental Health | 4 | 70.8 (17.5) | | 72 | | | 74.4 (20.4) | | | | | 38 | | 75.9 (19.7) | |  |  |
| Component summary scores | | |  | |  | | | |  |  | | | | |  |  |  |
| Physical (PCS-36) | 4 | 53.6 (9.3) | | 66 | | | 52.1 (10.6) | | | | | 34 | | 45.5 (8.7) | | |  |
| Mental (MCS-36) | 4 | 50.0 (8.4) | | 66 | | | 50.8 (9.7) | | | | | 34 | | 51.3 (9.3) | | |  |

SF-36: 36-item Short Form survey; PCS: physical component summary score; MCS: mental component summary score

^a^ Adjusted for age, sex, and chronic health conditions
^b^ Means and SD were estimated without bootstrapping, due to the limited sample sizes

Supplemental Table S5: **Estimated mean domain scores across PROMIS-29 domains, pain intensity item, and summary component scores across different bleeding phenotypes.** Note: because the means (SD) were estimated by re-running the bootstrapping procedure, small differences may be observed compared to the mean differences reported in Tables 5 and 6.

|  | Asymptomatic | | | Mild-to-moderate | | | | Severe | | | |
| --- | --- | --- | --- | --- | --- | --- | --- | --- | --- | --- | --- |
| PROMIS-29 domain | **n** | **Mean^a,b^ (SD)** | | **n** | | **Mean^a^ (SD)** | | **n** | | **Mean^a^ (SD)** | |
| Physical Function | 5 | 54.0 (7.7) | | 96 | | 51.6 (7.8) | | 46 | | 48.9 (8.8) | |
| Anxiety | 4 | 48.8 (8.6) | | 89 | | 50.1 (9.4) | | 43 | | 48.7 (8.5) | |
| Depression | 4 | 49.4 (7.9) | | 88 | | 47.8 (8.4) | | 39 | | 46.5 (7.5) | |
| Fatigue | 4 | 49.1 (9.5) | | 87 | | 48.4 (8.4) | | 40 | | 46.9(10.1) | |
| Sleep disturbance | 4 | 45.7 (7.9) | | 87 | | 47.3 (8.4) | | 40 | | 48.8 (9.5) | |
| Ability to Participate in Social Roles/ Activities | 4 | 55.5 (8.3) | | 85 | | 54.8 (9.2) | | 40 | | 52.1 (9.5) | |
| Pain Interference | 4 | 46.8 (8.5) | | 83 | | 48.1 (9.1) | | 39 | | 51.4 (8.7) | |
| Pain Intensity | 4 | 2.0 (2.4) | | 83 | | 1.8 (2.7) | | 39 | | 2.9 (2.5) | |
| Component summary scores | | |  | |  | |  | |  | |  |
| Physical (PCS-29) | 4 | 59.1 (11.9) | | 83 | | 52.3 (13.7) | | 39 | | 47.8 (13.7) | |
| Mental (MCS-29) | 4 | 53.8 (9.1) | | 83 | | 53.4 (10.0) | | 39 | | 53.1 (9.4) | |

PCS: physical component summary score; MCS: mental component summary score

^a^ Adjusted for age, sex, and chronic health conditions
^b^ Means and SD were estimated without bootstrapping, due to the limited sample sizes

Supplemental Table S6: **Cross-table of patient-reported severity and clinical bleeding phenotype.** In adults, Spearman’s rank correlation coefficient (rho = -0.314, p < .001) indicated a weak negative correlation between clinical bleeding phenotype and patient-reported severity.

|  |  | | Clinical bleeding phenotype | | |  |
| --- | --- | --- | --- | --- | --- | --- |
|  | **Patient-reported severity** | **Asymptomatic** | | **Mild-to-moderate** | **Severe** | |
| Adult | Not at all severe | 3 | | 22 | 5 | |
|  | Not severe | 2 | | 37 | 13 | |
|  | Rather severe | 0 | | 17 | 12 | |
|  | Severe | 0 | | 10 | 10 | |
|  | Very severe | 0 | | 1 | 3 | |
| Pediatric | Not at all severe | 1 | | 2 | 0 | |
|  | Not severe | 1 | | 6 | 5 | |
|  | Rather severe | 0 | | 4 | 0 | |
|  | Severe | 0 | | 1 | 1 | |
|  | Very severe | 0 | | 0 | 0 | |

Supplemental Table S7**. Estimated mean differences (MD) across SF-36 domains and summary component scores across patient subgroups.** Differences exceeding minimally important differences (MID) are highlighted in **bold**. An asterisk (*) indicates a significant difference (p≤ .006). Lower scores indicate worse outcomes.

|  |  | Severe phenotype without (n=33 to 30) vs with prophylaxis (n=8 to 6) | Bi-allelic (n= 29 to 23) vs heterozygous recessive genotype (n=55 to 46) | Diagnosed due to symptoms (n=54 to 46) vs due to family member (n= 62 to 51) |
| --- | --- | --- | --- | --- |
| SF-36 domain | **MID** | **MD^a^ (99.4% CI)** | **MD^a^ (99.4% CI)** | **MD^a^ (99.4% CI)** |
| Physical functioning | **≥5** | **4.6 (-29.9, 47.0)** | -4.1 (-19.4, 9.7) | -4.9 (-15.9, 7.5) |
| Role limitation due to physical health | **≥5** | **-15.5 (-63.1, 61.2)** | -4.3 (-27.8, 16.3) | **-8.2 (-26.5, 10.1)** |
| Bodily pain | **≥5** | **-10.6 (-37.4, 21.6)** | **-7.4 (-24.0, 9.4)** | **-7.8 (-21.1, 6.0)** |
| General health | **≥5** | **-12.2 (-41.8, 28.9)** | -4.2 (-21.8, 8.8) | -4.0 (-15.2, 8.0) |
| Vitality | **≥5** | **-16.5 (-38.4, 7.3)** | 1.2 (-10.0, 12.2) | 0.7 (-8.3, 9.6) |
| Social functioning | **≥5** | **-11.6 (-36.8, 18.1)** | **-5.0 (-17.0, 7.4)** | **-5.4 (-14.8, 5.1)** |
| Role limitations due to emotional problems | **≥5** | **-11.1 (-43.7, 19.9)** | -0.8 (-13.8, 13.8) | **-13.9* (-28.7, -0.0)** |
| Mental health | **≥5** | **-16.6 (-43.6, 4.6)** | **6.4 (-7.0, 16.6)** | 3.4 (-5.1, 12.7) |
| Component summary scores | |  |  |  |
| Physical (PCS-36) | **≥2** | -2.1 (-15.2, 14.4) | **-4.4 (-12.3, 2.6)** | **-3.0 (-8.1, 2.6)** |
| Mental (MCS-36) | **≥3** | **-7.1 (-14.6, 0.8)** | **3.6 (-1.2, 8.5)** | -1.0 (-5.8, 4.5) |

SF-36: 36-item Short Form survey; HMB: heavy menstrual bleeding; RBD: rare bleeding disorder, MID: minimally important difference; PCS: physical component summary score; MCS: mental component summary score
^a^ Adjusted for age, sex, and chronic health conditions

Supplemental Table S8**. Estimated mean differences (MD) across PROMIS-29 domains, the pain intensity item, and summary component scores across patient subgroups.** Differences exceeding minimally important differences (MID) are highlighted in **bold**. An asterisk (*) indicates a significant difference (p≤ .006). A higher score indicates more of the construct.

|  |  | Severe phenotype without (n=34 to 30) vs with prophylaxis (n=12 to 9) | Bi-allelic (n= 37 to 32) vs heterozygous recessive genotype (n=61 to 54) | Diagnosed due to symptoms (n=64 to 57) vs affected family member (n= 65 to 57) |
| --- | --- | --- | --- | --- |
| PROMIS-29 domain | **MID** | **MD^a^ (99.4% CI)** | **MD^a^ (99.4% CI)** | **MD^a^ (99.4% CI)** |
| Physical function | **≥-2** | **-3.7 (-11.7, 5.3)** | -1.6 (-6.0, 3.5) | **-2.2 (-6.2, 1.6)** |
| Anxiety | **≥2.3** | **4.3 (-3.8, 11.5)** | -0.8 (-5.7, 4.3) | -0.6 (-4.7, 4.0) |
| Depression | **≥3.0** | **6.0 (-0.2, 13.2)** | -0.9 (-5.2, 3.8) | -1.4 (-5.5, 2.9) |
| Fatigue | **≥2** | **9.3 (-0.1, 21.0)** | 0.3 (-5.5, 6.7) | 0.4 (-4.1, 5.6) |
| Sleep disturbance | **≥1** | **8.9 (-4.1, 19.9)** | -0.6 (-5.6, 4.4) | **2.1 (-1.9, 6.1)** |
| Ability to participate in  social roles/activities | **≥-1** | **-4.0 (-14.9, 5.9)** | **-2.4 (-7.3, 2.8)** | -0.9 (-5.3, 3.3) |
| Pain interference | **≥2.0** | **2.4 (-6.2, 14.0)** | 1.8 (-3.2, 7.1) | 1.6 (-2.8, 6.4) |
| Pain intensity | **≥1** | 0.6 (-1.5, 3.5) | 0.5 (-0.9, 2.0) | 0.7 (-0.5, 2.0) |
| Component summary scores | | |  |  |
| Physical health (PCS-29) | **≥6.2** | -5.7 (-21.5, 14.9) | -2.9 (-10.5, 5.6) | -2.1 (-8.0, 4.2) |
| Mental health (MCS-29) | **≥4.7** | **-9.1* (-20.2, -1.1)** | -0.7 (-6.5, 4.8) | -0.5 (-5.5, 4.5) |

PROMIS-29: Patient-reported Measurement Information System, Profile 29; HMB: heavy menstrual bleeding; RBD: rare bleeding disorder, MID: minimally important difference; PCS: physical component summary score; MCS: mental component summary score

^a^ Adjusted for age, sex, and chronic health conditions

**References**

1 Statistiek CBvd. Gezonde levensverwachting; vanaf 1981. 2024.

2 Elsman EBM, Flens G, de Beurs E, Roorda LD, Terwee CB. Towards standardization of measuring anxiety and depression: Differential item functioning for language and Dutch reference values of PROMIS item banks. *PLoS One*. 2022; **17**: e0273287. 10.1371/journal.pone.0273287.

3 Elsman EBM, Roorda LD, Crins MHP, Boers M, Terwee CB. Dutch reference values for the Patient-Reported Outcomes Measurement Information System Scale v1.2 - Global Health (PROMIS-GH). *J Patient Rep Outcomes*. 2021; **5**: 38. 10.1186/s41687-021-00314-0.

4 Terwee CB, Elsman EB, Roorda LD. Towards standardization of fatigue measurement: Psychometric properties and reference values of the PROMIS Fatigue item bank in the Dutch general population. *Research Methods in Medicine & Health Sciences*. 2022; **3**: 86-98. 10.1177/26320843221089628.

5 Terwee CB, Roorda LD. Country-specific reference values for PROMIS((R)) pain, physical function and participation measures compared to US reference values. *Ann Med*. 2023; **55**: 1-11. 10.1080/07853890.2022.2149849.

6 Terwee CB, van Litsenburg RRL, Elsman EBM, Roorda LD. Psychometric properties and reference values of the Patient-Reported Outcomes Measurement Information System (PROMIS) sleep item banks in the Dutch general population. *J Sleep Res*. 2023; **32**: e13753. 10.1111/jsr.13753.

7 Aaronson NK, Muller M, Cohen PD, Essink-Bot ML, Fekkes M, Sanderman R, Sprangers MA, te Velde A, Verrips E. Translation, validation, and norming of the Dutch language version of the SF-36 Health Survey in community and chronic disease populations. *J Clin Epidemiol*. 1998; **51**: 1055-68. 10.1016/s0895-4356(98)00097-3.
